# Rapid Recovery From Bell's Palsy Using Transcranial Magnetic Stimulation of the Facial Nerve: A Case Report

**DOI:** 10.7759/cureus.77721

**Published:** 2025-01-20

**Authors:** Nguyen L Ngo, Maher Asfour, Kayla Tran, Gordon Cheung, Thinh Mai

**Affiliations:** 1 Medicine, Kansas City University, Joplin, USA; 2 Medicine, Kansas City University, Kansas City, USA; 3 Psychiatry, South Bay Behavioral Health, Los Gatos, USA

**Keywords:** bell's palsy, case report, facial paralysis, non-invasive procedure, transcranial magnetic stimulation

## Abstract

Transcranial magnetic stimulation (TMS), a non-invasive tool that uses magnetic fields to stimulate specific regions within the brain, has emerged as a versatile treatment modality in both research and clinical settings. While its utilization in psychiatry for treatment-resistant depression is well established, TMS is increasingly gaining traction for its use in diverse neurological conditions, including idiopathic facial nerve palsy, post-stroke rehabilitation, autism spectrum disorder, and hereditary ataxia. Through its ability to trigger neuronal plasticity and potentiate synaptic transmission, it is able to provide significant therapeutic potential. This paper seeks to explore and add to the rising research in treating idiopathic facial nerve palsy with the use of peripheral TMS. A 26-year-old woman with no prior history of facial palsy or related conditions presented with acute-onset left-sided facial paralysis upon awakening, following a strenuous hiking trip the previous day. Based on the modified House-Brackmann scale, she was determined to have grade V facial paralysis (severe facial weakness with barely perceptible motion). After 10 treatments over the course of two weeks, the patient's facial paralysis improved to grade III (obvious, moderate facial weakness, complete eye closure with maximal effort, and good forehead movement). At one-week post-TMS treatment, the patient reported full recovery to all facial expressions and no adverse effects were noted. This case report aims to show the effectiveness of utilizing TMS as a treatment option for idiopathic facial nerve palsy.

## Introduction

Bell's palsy is a type of unilateral facial palsy caused by a lesion to the seventh cranial nerve. It accounts for up to 83% of cases of facial paralysis [1]. Diagnosis of Bell's palsy requires the exclusion of all other acute-onset facial paralysis. Clinical symptoms and degrees of severity vary from patients, but the common ones include impaired mobility of the eyelid, forehead, and corner of the mouth on the affected side, resulting in potential dry eyes and drooling. Others may experience numbness of the tongue and cheek [2]. While the cause of the disease is not apparent, the idiopathic facial dysfunction has been associated with viruses like human herpesvirus and cytomegalovirus [1]. One of the ways to quantify the severity of a patient's facial paralysis is using the modified House-Brackmann scale. This standardized tool evaluates facial nerve function with specific facial regional analysis, providing a thorough measurement [3]. 

Early management with corticosteroids should be commenced within the first 72 hours of onset [4]. Antivirals may be administered as a combination with steroids if the etiology is suspected to be herpes zoster or varicella-zoster virus [2]. Most patients recover completely, but those who do not may experience facial contractions that require long-term treatment with botulinum toxin injections with possible need for surgery [1]. Less conventional treatments have also been shown to improve facial nerve function. One study found patients who were treated with electrical stimulation showed more facial nerve function improvement than the control group [5].

Transcranial magnetic stimulation (TMS) is a type of treatment that uses magnetic fields to induce electrical currents in the brain through neuronal plasticity [6]. The treatment is non-invasive and has few side effects, making it a favorable treatment for patients. It is currently being used for treatment-resistant depression, where the electrical currents target the left dorsolateral prefrontal cortex of the brain [7]. Additionally, TMS is being studied to be used as a treatment option in autism spectrum disorder, post-stroke rehabilitation, and hereditary ataxia [7-9]. As of recent, few studies have attempted to treat facial paralysis with TMS [10,11]. In this case report, we present a patient with idiopathic Bell's palsy who was treated with TMS and aim to illustrate the promising results of using this treatment for those affected by facial nerve palsy.

## Case presentation

The patient was a 26-year-old woman with a past medical history of chronic hives who experienced a gradual onset of left facial paralysis on September 1, 2024, following a strenuous hiking trip. She reported the duration of the hike was four hours with no complaint of exposure to trauma, bacteria, or viruses. She was diagnosed with idiopathic Bell's palsy on September 2, 2024, at Action Urgent Care. The absence of a vesicular rash around the ear and the lack of an erythema migrans lesion or parotid gland swelling effectively ruled out Ramsay Hunt syndrome, Lyme disease, and sarcoidosis as differential diagnoses. Her left facial paralysis worsened from a grade I (normal facial function) to a grade V (severe facial nerve palsy) within one day, measured using the modified House-Brackmann grading scale [12]. The patient suffered from an inability to close her left eyelid and used Systane eye drops every two hours to help with dry eye symptoms. Additionally, she had an inability to control the left side of her face including raising the ipsilateral forehead, moving the corner of her mouth, and puffing out her cheek and also had partial numbness of the tongue. The patient initiated the treatment within the first 48 hours and completed a course of prednisone 20 mg, four tablets once daily for seven days which she started on September 3, 2024, from the urgent care center. The patient did not transition into a tapering phase of her steroid treatment as the prescribing provider deemed it unnecessary. She was also prescribed doxycycline 100 mg twice a day for 10 days but terminated the medical regimen after two days due to side effects of nausea and vomiting. While the patient did not mention any recent tick bite that could result in Lyme disease, the provider recommended doxycycline as a prophylaxis due to the prevalence of ticks in wooded areas. No other alternative treatments were prescribed. 

She was not taking any medications other than the prescribed prednisone and doxycycline. She has no past surgeries, no physical trauma to the face, and no pertinent family history. The patient is a student who denies smoking but consumes two five-ounce glasses of wine per month. Physical examinations revealed an alert and oriented ×4 patient with the following muscles experiencing complete or partial paralysis on the left side of her face: the frontalis, orbicularis oculi, zygomaticus major and minor, buccinator, orbicularis oris, depressor anguli oris, levator labii superioris, depressor labii inferioris, mentalis, and platysma. A thorough review of all systems showed no other abnormalities. No lab investigations were conducted. 

The patient presented to South Bay Behavioral Health clinic, operated by Dr. Thinh Mai, on September 8, 2024, as an outpatient with no reduction in symptoms. The patient was offered treatment for her Bell's palsy using TMS. After presenting the risks (potential seizure, headache, and discomfort), benefits, and alternatives of the TMS procedure to the patient, an informed consent was obtained on the same day. The patient officially began TMS treatments on September 9, 2024, eight days after the onset of symptoms without any additional or continuation of medications.

Procedure

**Figure 1:**
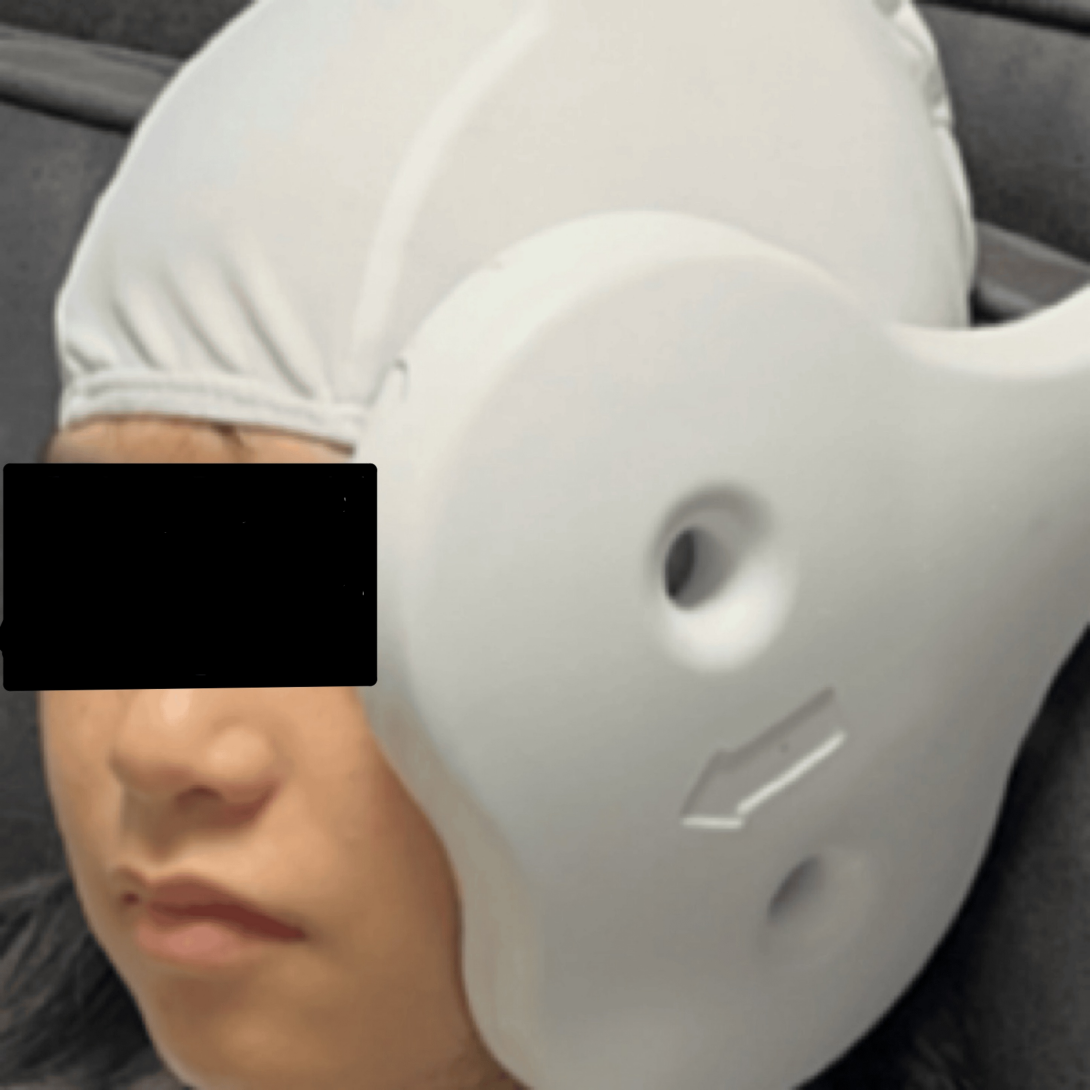
The experimental setup demonstrating the placement of figure-eight coil on the patient

The TMS protocol used on this patient was adapted from a previously published method [8]. The patient was placed in a Fowler's position and her head was covered with a cloth liner cap. The TMS instrument used was the CloudTMS machine (Neurosoft, Ivanovo, Russia). The center of the machine's figure-eight coil was fixed on the lower margin of the zygomatic arch and the mandibular notch on the left side of the patient's face (Figure 1). The instrument parameters were set at 5.0 Hz stimulation frequency for six seconds, with a 14-second rest period between trains. These settings delivered a total of 60 trains (1,800 pulses) during the course of a 20-minute session. The patient sat through this session one time per day, five times per week. The treatment lasted for a total of 12 days and officially concluded on September 20, 2024. 

Results

**Figure 2:**
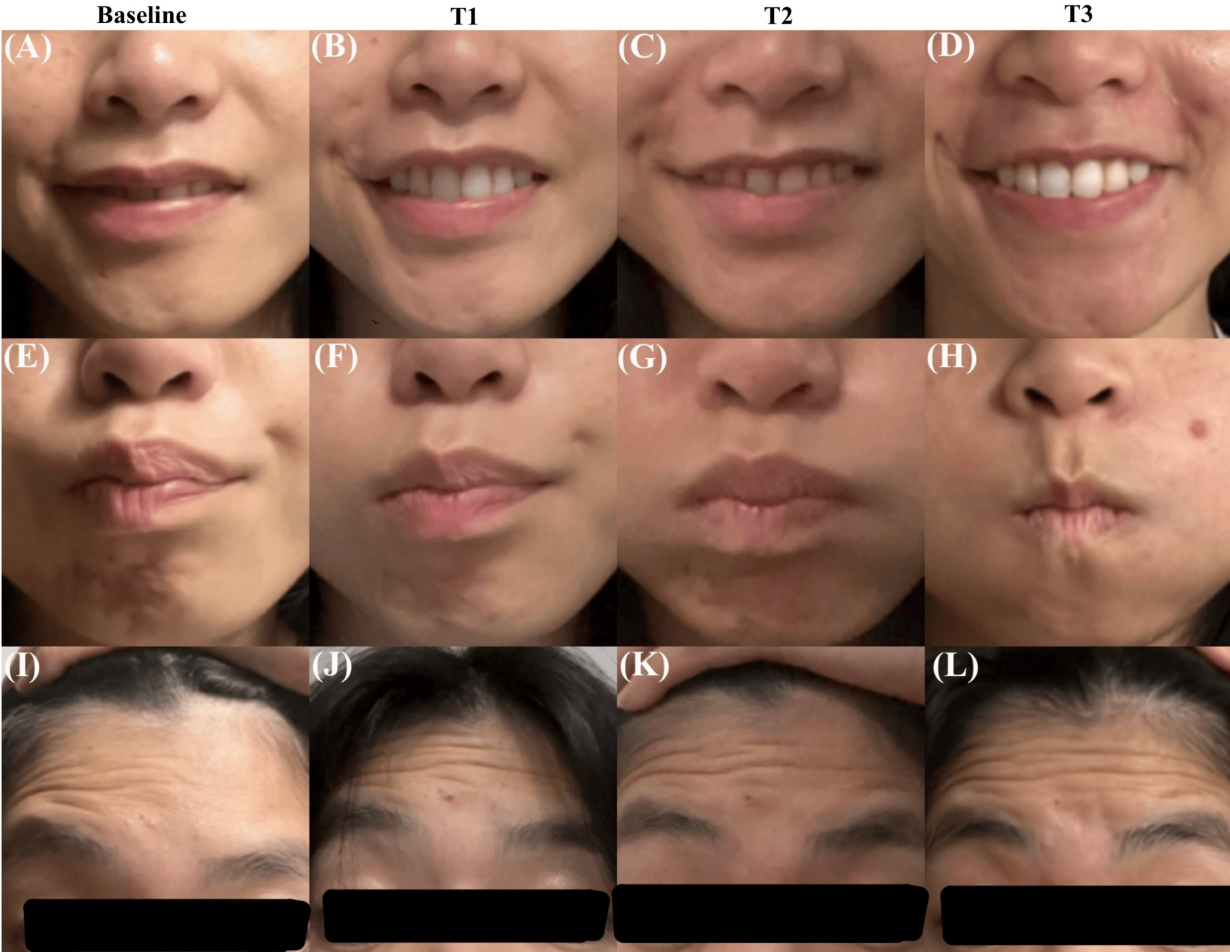
Photos capture the outward movement of the mouth (A-D), nasolabial weakness (E-H), and upward movement of the eyebrow and forehead (I-L). Sequential images document the facial movement improvement at the initial presentation (baseline), the first week of treatment (T1), the second week of treatment (T2), and one-week post-treatment (T3)

Symptom progression was documented and graded using the modified House-Brackmann grading scale after the first week of treatment (T1), the second week of treatment (T2), and one-week post-treatment completion (T3) (Table 1). The patient did not receive additional therapies alongside TMS. Prior to TMS treatment (baseline), the patient's facial paralysis was scored a grade V on the House-Brackmann scale (Figure 2A, Figure 2E, and Figure 2I). She had a partially disfigured face with a complete inability to move the oral area. Very slight movement was noted when she attempted to raise her eyebrows or close her eyelids. After one week of treatment (T1), the patient's facial paralysis was reduced to a grade IV (Figure 2B, Figure 2F, and Figure 2J). She showed an improved ability to raise her eyebrows and move her eyelids, yet it was not enough to close her eyelid. Trace movement was noticed around the oral area. After the second week of treatment (T2), the patient's facial paralysis was reduced to a grade III (Figure 2C, Figure 2G, and Figure 2K). Significant improvement was observed in eyelid movement, capable of full closure of the eyelid with maximal effort. Improvements were also seen with eyebrow elevation and oral movement (Figure 2D, Figure 2H, and Figure 2L). After one-week post-TMS treatment, the patient reported full recovery (grade I) to all facial expressions, and no adverse effects were noted. During the final follow-up at two weeks post-treatment, the patient reported her condition and functional status remained unchanged from the last follow-up.

**Table 1:** Modified House-Brackmann results before and after treatment The table records the scores of facial function at the initial presentation (baseline), the first week of treatment (T1), the second week of treatment (T2), and one-week post-treatment (T3). Higher scores from each category correspond to progressively severe impairment. The modified House-Brackmann scale is graded as follows: grade I (normal symmetric facial function), grade II (slight dysfunction), grade III (moderate dysfunction), grade IV (moderately severe dysfunction), grade V (severe dysfunction), and grade VI (total paralysis)

Time	Eye score	Eyebrow score	Nasolabial fold score	Oral score	Total score	Grade
Baseline	5	5	6	6	22	V
T1	4	3	5	5	17	IV
T2	2	2	4	2	10	III
T3	1	1	1	1	4	I

## Discussion

To the best of our knowledge, this is the first case study in the United States to explore TMS as an adjuvant therapy for Bell's palsy, with results suggesting that TMS could be a promising alternative treatment for this condition. The patient, who initially presented with severe facial paralysis (grade V on the modified House-Brackmann scale), demonstrated rapid improvement in facial muscle control to a grade III throughout the two-week course of TMS. One week after the treatment was finished, the patient demonstrated full recovery, including the ability to fully close her eyelid and move her oral area. This reflects a complete recovery achieved in less than a month from symptom onset. This outcome is noteworthy, as according to a randomized controlled trial performed by Engström et al., the median time for complete recovery with early steroid treatment is 75 days [13].

Previous studies have shown that both electrical and magnetic stimulation can accelerate the recovery of damaged nerve function by supporting the development of new synaptic connections and enhancing axonal regeneration [10,14,15]. However, the exact mechanism as to how TMS assists in the recovery of Bell's palsy is not well understood. Some studies suggest that TMS causes an increase in local blood flow, providing the damaged nerve with the necessary nutrients it needs to recover and reduce symptoms [10,16,17]. A previous study suggests that the mechanical vibrations that arise from TMS-induced muscle contractions strengthen the damaged nerve's control over the facial muscle [18]. The actual mechanism behind TMS's effectiveness in treating Bell's palsy could be a combination of these proposed effects. Contraindications and precautions for the use of TMS must be taken into account, including ferromagnetic or conductive implants near the TMS coil, unstable or uncontrolled medical conditions, an active high-risk seizure focus, a history of seizures or epilepsy, medications that lower the seizure threshold, certain neurological conditions, pregnancy, ongoing substance use, severe psychiatric conditions, other implanted devices, and significant cardiovascular or other systemic conditions [19]. The only current FDA-approved use of TMS is for the treatment of major depressive disorder in adults aged 18 and older; however, several studies have investigated its use in both younger and older populations for various applications [20].

Future studies should investigate the potential of TMS as a mainstream treatment option for Bell's palsy by conducting randomized controlled trials with large sample sizes to assess its efficacy and safety. It would also be beneficial to compare different TMS parameters (such as frequency and dosage), to determine the optimal settings for recovery. Such studies could allow for more individualized and effective treatment plans for patients with facial nerve paralysis. Moreover, TMS could be explored as a non-invasive facial rehabilitation for other causes of facial paralysis such as Ramsay Hunt syndrome, Lyme disease, or sarcoidosis.

## Conclusions

This case study demonstrates the potential of using TMS as a promising therapeutic option for Bell's palsy. The patient's rapid and complete recovery following a relatively short course of TMS suggests that this non-invasive intervention would make it worthwhile to continue exploring TMS as a treatment option in conjunction with standard glucocorticoid therapy, as a standalone therapy, and also for other causes of facial paralysis such as Ramsay Hunt syndrome, Lyme disease, and sarcoidosis. Future research with larger samples and different treatment parameters, such as intensity, frequency, localization, and total pulses, will be necessary to validate the efficacy and safety of TMS and explore optimal treatment synergies and modalities utilizing TMS for facial nerve palsies. To the best of our knowledge, this is the first case study in the United States to explore the utilization of TMS as an adjuvant therapy for Bell's palsy.
